# Effects of age on dynamic balance measures and their correlation during walking across the adult lifespan

**DOI:** 10.1038/s41598-022-18382-7

**Published:** 2022-08-22

**Authors:** Takeshi Yamaguchi, Kei Masani

**Affiliations:** 1 Department of Finemechanics, Graduate School of Engineering, Tohoku University, 6-6-01 Aramaki-Aza-Aoba, Aoba-ku, Sendai, Miyagi 980-8579 Japan; 2 Graduate School of Biomedical Engineering, Tohoku University, 6-6-01 Aramaki-Aza-Aoba, Sendai, Miyagi 980-8579 Japan; 3 Institute of Biomedical Engineering, University of Toronto, 164 College Street, Toronto, ON M5S 3G9 Canada; 4 KITE Research Institute, Toronto Rehabilitation Institute—University Health Network, Toronto, ON M4G 3V9 Canada

**Keywords:** Geriatrics, Biomedical engineering, Mechanical engineering

## Abstract

In this study, we aimed to discover (1) the effects of age on dynamic balance measures, including the margin of stability (MOS), whole-body angular momentum (*H*), and misalignment of the desired and measured centers of pressure (dCOP and mCOP, respectively) in the anteroposterior (AP) and mediolateral (ML) directions, (2) the relationship between gait parameters and these balance measures, and (3) the relationships between these balance measures. We used the kinetic and kinematic data of 151 participants aged 20–77 years from a publicly available database. Participants were divided into three groups: young, middle-aged, and old. The step width of the old group was higher than that of the young group. Age-related differences in dynamic measures were found in the ML direction and not in the AP direction: MOS, peak-to-peak range of *H*, and dCOP–mCOP in the old group were greater than in the young group. ML MOS positively correlated with the frontal peak-to-peak range of *H.* The ML peak-to-peak range of *H* positively correlated with ML dCOP–mCOP across the adult lifespan. Our findings provide new insights for understanding the effects of age on dynamic balance and the relationships between the metrics. Older adults walked with a larger step width, resulting in a large stability margin in the ML direction, although with increased moment and momentum around the center of mass in the frontal plane.

## Introduction

The frequent occurrence of falls is a serious problem in older adults. It suggests that dynamic balance during walking deteriorates due to aging in multiple physiological systems, such as muscular and sensory systems^1–3^. Various metrics have been proposed for evaluating dynamic balance during walking, such as stride-to-stride variability (e.g., standard deviation, coefficients of variation of gait parameters)^4–8^, margin of stability (MOS)^9–12^, and whole-body angular momentum (*H*)^13–15^. We have previously proposed misalignment between the desired center of pressure (dCOP) and the measured center of pressure (mCOP) as a measure of dynamic balance^16^.

MOS is the minimum distance between the extrapolated center of mass (XCOM) and the border of the base of support (BOS), which should be positive (i.e., the BOS should capture the XCOM) to maintain balance during walking^9^. This measure accounts for the body’s center of mass (COM) position and its velocity with respect to foot placement. MOS has been studied in younger and older adults during walking^17^, stepping on targets^18^, as well as during head turning while walking^19^, indicating that the MOS in the mediolateral (ML) direction for older adults increased compared with that for younger adults due to a wider foot placement. The value of *H* needs to be within a certain degree to maintain smooth walking^13^. Previous research has indicated that a substantial change in frontal plane *H* during the gait cycle is associated with poorer clinical balance scores in post-stroke individuals^15^. Vistamehr and Neptune^20^ reported that older adults increased their range of *H* in the frontal plane compared with younger adults, and that this increase was associated with the increased external moment arm in the ML direction in older adults, which correlated to the range of frontal *H*. The dCOP is a virtual point on the ground equivalent to the centroid moment pivot^13,21^ and target zero-moment point^22^ that was proposed in bipedal robotics. The external moment about the body COM is zero when the actual center of pressure (i.e., mCOP) is coincident with the dCOP^16^. The dCOP–mCOP value is proportional to the external moment about the COM; an increase in dCOP–mCOP results in increased external moment about the COM, potentially leading to the loss of postural balance. Yamaguchi et al. investigated dCOP–mCOP in a turning gait on a slippery floor and found that dCOP–mCOP in the lateral direction is a good indicator of falling^16^.

We could expect these balance measures to identify aging effects on dynamic balance during walking. However, several previous studies have investigated the effect of age on the dynamic balance measures using a small number of samples^17–20^, and therefore, have not investigated the effect of age as a continuous variable on the dynamics measures. In addition, to date, no study has investigated the effects of aging on gait by using all three of these balance measures simultaneously, and no comparison has been made across the three balance measures for a large sample data across the adult lifespan. This study aimed to investigate the effects of age on MOS, degree of *H*, and dCOP–mCOP during walking, the relationship between gait parameters and these balance measures, and the relationships between the measures across the adult lifespan using a publicly available gait database, which included 151 participants aged 20–77 years^23^. Previous studies have suggested that balance control in the ML direction is more challenging than that in the anteroposterior (AP) direction and that active control is needed to regulate balance in the ML direction^24,25^. Furthermore, older adults have difficulty in controlling balance in the ML direction, which is of crucial importance to prevent lateral falls that increase the risk of debilitating hip fracture injury^26,27^. Thus, we hypothesized that aging effects in the abovementioned measures would be more prominent in the ML direction (frontal plane) than in the AP direction (sagittal plane) during walking. We also hypothesized that age-related changes in the gait parameters affect the balance measures.

## Results

**Figure 1:**
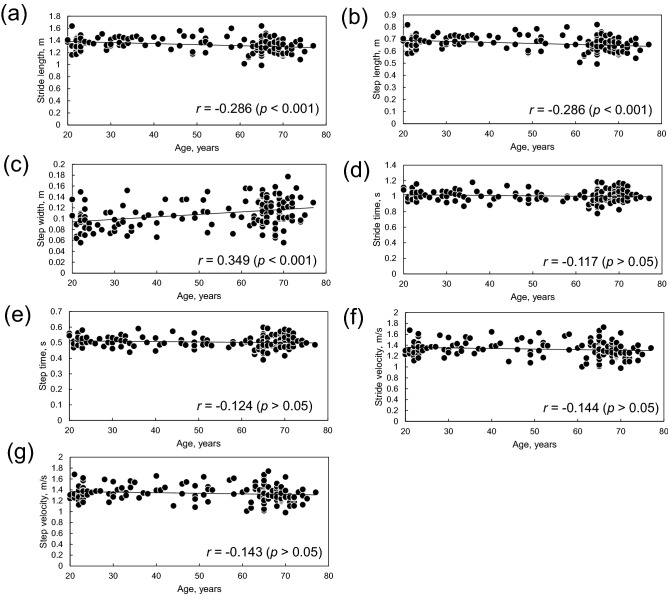
Relation between age and (**a**) stride length, (**b**) step length, (**c**) step width, (**d**) stride time, (**e**) step time, (**f**) stride velocity, and (**g**) step velocity.

**Figure 2:**
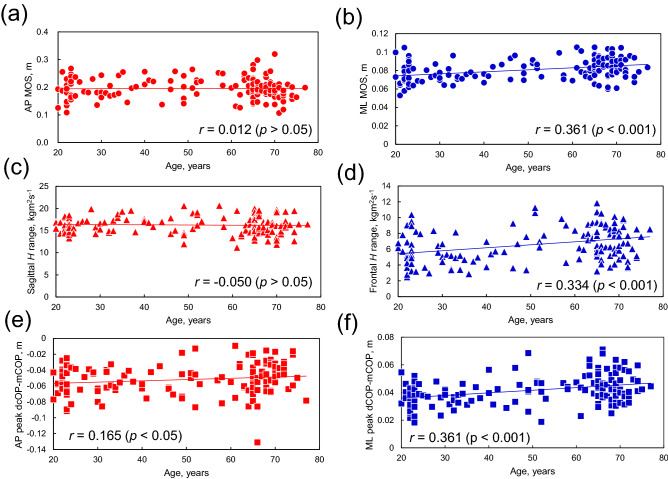
Relation between age and dynamic balance measures: (**a**) anteroposterior (AP) margin of stability (MOS), (**b**) mediolateral (ML) MOS, (**c**) sagittal *H* range, (**d**) frontal *H* range, (**e**) AP peak desired center of pressure–measured center of pressure (dCOP–mCOP), and (**f**) ML peak dCOP–mCOP.

Figure 1 shows the relationship between age and gait parameters. Among gait parameters, step width had a weak positive correlation with age (*r* = 0.349, *p* < 0.001, Fig. 1c). Figure 2 shows the relation between age and dynamic balance measures in the AP and ML directions. No correlation was found between age and dynamic balance measures in the AP directions (*r* < 0.2, *p* > 0.05, Fig. 2a). Dynamic balance measures in the ML directions had a weak positive correlation with age (ML MOS: *r* = 0.361, *p* < 0.001, Fig. 2b; frontal *H* range: *r* = 0.334, *p* < 0.001, Fig. 2d; ML peak dCOP–mCOP: *r* = 0.361, *p* < 0.001, Fig. 2f).

Table 1 summarizes the results of the gait parameters and dynamic balance measures for all the age groups. One-way analysis of covariance (ANCOVA) indicated that step width (*F*[2,147] = 12.813, *p* < 0.001), stride time (*F*[2,147] = 4.981, *p* = 0.008), and step time (*F*[2,147]  = 4.924, *p* = 0.009) were significantly affected by age group. The post-hoc *t*-test revealed that the step width for the old group (*p* < 0.001, Cohen’s *d* = 0.837) and middle-aged group (*p* = 0.031, Cohen’s *d* = 0.636) were significantly greater than for the young group. Stride and step times in the middle-aged group were shorter than that in the young (*p* = 0.036, Cohen’s *d* = 0.188) and old (*p* = 0.009, Cohen’s *d* = 0.430) groups. No significant age group effects were found for the other gait parameters (*p* > 0.05).

**Table 1:** Summary of ANOVA, ANCOVA, and post-hoc *t*-test for gait parameters and dynamic balance measures.

	YA		MA		OA		ANOVA	ANCOVA	Post-hoc *t*-test
	Mean	SD	Mean	SD	Mean	SD			
**Gait parameters**									
Stride length, m	1.361	0.094	1.340	0.136	1.296	0.111	–	NS	–
Step length, m	0.681	0.047	0.670	0.068	0.648	0.056	–	NS	–
Step width, m	0.096	0.022	0.110	0.022	0.116	0.025	–	*p* < 0.001	YA-MA, *p* < 0.05; YA-OA, *p* < 0.001
Stride velocity, m/s	1.344	0.128	1.361	0.161	1.300	0.144	NS	–	–
Step velocity, m/s	1.351	0.129	1.368	0.162	1.307	0.145	NS	–	–
Stride time, s	1.017	0.060	0.989	0.071	1.003	0.082	–	*p* < 0.01	YA-MA, *p* < 0.05; MA-OA, *p* < 0.01
Step time, s	0.511	0.030	0.497	0.035	0.504	0.041	–	*p* < 0.01	YA-MA, *p* < 0.05; MA-OA, *p* < 0.01
**Dynamic balance measures**									
AP MOS, m	0.192	0.037	0.207	0.038	0.193	0.039	NS	–	–
ML MOS m	0.075	0.010	0.081	0.010	0.085	0.011	–	*p* < 0.001	MA-OA, *p* < 0.01; YA-OA, *p* < 0.001
*H* range (sagittal plane), kg m^2^s^−1^	16.243	1.454	16.444	2.298	16.178	1.885	–	NS	–
*H* range (frontal plane), kg m^2^s^−1^	5.670	2.082	6.574	2.205	6.964	1.992	–	*p* < 0.001	MA-OA, *p* < 0.01; YA-OA, *p* < 0.001
AP peak dCOP–mCOP, m	− 0.055	0.017	− 0.053	0.020	− 0.048	0.019	NS	–	–
ML peak dCOP–mCOP, m	0.037	0.008	0.043	0.011	0.045	0.009	*p* < 0.001	–	YA-MA, *p* < 0.05; YA-OA, *p* < 0.001

*ANCOVA* one way analysis of covariance; *ANOVA* one way analysis of variance; *AP* anteroposterior; *dCOP* desired center of pressure; *MA* middle-aged adult group; *mCOP* measured center of pressure; *ML* mediolateral; *MOS* margin of stability; *NS* no significance; *OA* old adult group; *YA* young adult group.

The MOS (one-way ANCOVA, *F*[2,147] = 20.067, *p* < 0.001), peak-to-peak range of *H* (one-way ANCOVA, *F*[2,147] = 15.054, *p* < 0.001), and peak dCOP–mCOP (one-way analysis of variance [ANOVA], *F*[2,148] = 10.269, *p* < 0.001) in the ML direction were significantly affected by the age groups. The post-hoc *t*-test indicated that the ML MOS in the old group was greater than in the young (*p* < 0.001, Cohen’s *d* = 0.909) and middle-aged (*p* = 0.003, Cohen’s *d* = 0.374) groups. The peak-to-peak range of *H* in the frontal plane in the old group was greater than that in the young (*p* < 0.001, Cohen’s *d* = 0.638) and middle-aged (*p* = 0.005, Cohen’s *d* = 0.189) groups. The ML peak dCOP–mCOP in the old (*p* < 0.001, Cohen’s *d* = 0.927) and middle-aged (*p* = 0.013, Cohen’s *d* = 0.633) groups was significantly greater than that in the young group. The dynamic balance measures in the AP direction were not affected by the age groups (*p* > 0.05).

Stepwise multiple regression analysis revealed that age, body height, body mass, and gait parameters explain 87.1% and 72.5% of AP MOS and ML MOS, respectively (Table 2). The standardized regression coefficient indicated that AP MOS was significantly affected by step length and velocity and that ML MOS was strongly affected by step width. Furthermore, both AP and ML MOS were positively correlated with age. In contrary, the adjusted coefficients of determination for the other dynamic balance measures (peak-to-peak range of *H* and peak dCOP–mCOP) were lower (*R*^2^_adj_ ≤ 0.424); however, the peak-to-peak range of *H* and peak dCOP–mCOP in the ML direction showed a positive correlation with age.

**Table 2:** Standardized regression coefficient of each independent variable (age, body height, body mass, and gait parameters) and adjusted coefficient of determination for stepwise multiple regression analysis for each dynamic stability measure.

	Age	Body height	Body mass	Stride length	Step length	Step width	Stride velocity	Step velocity	Stride time	Step time	R^2^_adj_
AP MOS	0.081*	0.236***	–	–	− 0.521***	0.069*	–	1.275***	–	–	0.871
ML MOS	0.108*	0.199**	0.207***	–	− 0.140**	0.660***	–	–	–	–	0.725
*H* range (sagittal plane)	–	–	0.360***	–	0.503***	–	–	–	–	–	0.424
*H* range (frontal plane)	0.300***	0.215*	0.232**	–	–	0.233**	–	0.183**	–	–	0.372
AP peak dCOP–mCOP		–	–	–	–	–	− 0.456***	–	–	–	0.202
ML peak dCOP–mCOP	0.449***	0.280**	–	–	–	–	–	0.306**	− 0.216*	–	0.356

*AP* anteroposterior; *dCOP* desired center of pressure; *mCOP* measured center of pressure; *ML* mediolateral; *MOS* margin of stability.

*p < 0.05; **p < 0.01; ***p < 0.001.

Table 3 shows the results of the partial correlation analysis among the dynamic balance measures. In the AP direction, the partial correlation coefficient was > 0.2 between the dynamic balance measures (*r* =  0.202, *p* = 0.013 between the MOS and peak-to-peak range of *H*, corrected for peak dCOP–mCOP; *r* =  − 0.350, *p* < 0.001 between the MOS and peak dCOP–mCOP, corrected for peak-to-peak range of *H*; and *r* = − 0.225, *p* = 0.006 between the peak dCOP–mCOP and peak-to-peak range of *H*, corrected for MOS). In the ML direction, a positive correlation with a strong partial correlation coefficient (> 0.3) was found between the MOS and peak-to-peak range of *H*, corrected for peak dCOP–mCOP (*r* = 0.475, *p* < 0.001) and between peak dCOP–mCOP and peak-to-peak range of *H*, corrected for MOS (*r* = 0.353, *p* < 0.001).

**Table 3:** Summary of partial correlation analysis among dynamic balance measures in the AP and ML directions.

Balance measure 1	Balance measure 2	Control variable	Partial correlation coefficient
*H* range (sagittal plane)	AP MOS	AP peak dCOP–mCOP	0.202*
AP peak dCOP–mCOP	AP MOS	*H* range (sagittal plane)	− 0.350***
*H* range (sagittal plane)	AP peak dCOP–mCOP	AP MOS	− 0.225**
*H* range (frontal plane)	ML MOS	ML peak dCOP–mCOP	0.475***
ML peak dCOP–mCOP	ML MOS	*H* range (frontal plane)	0.073
*H* range (frontal plane)	ML peak dCOP–mCOP	ML MOS	0.353***

*AP* anteroposterior; *dCOP* desired center of pressure; *mCOP* measured center of pressure; *ML* mediolateral; *MOS* margin of stability.

*p < 0.05; **p < 0.01; ***p < 0.001.

## Discussion

**Figure 3:**
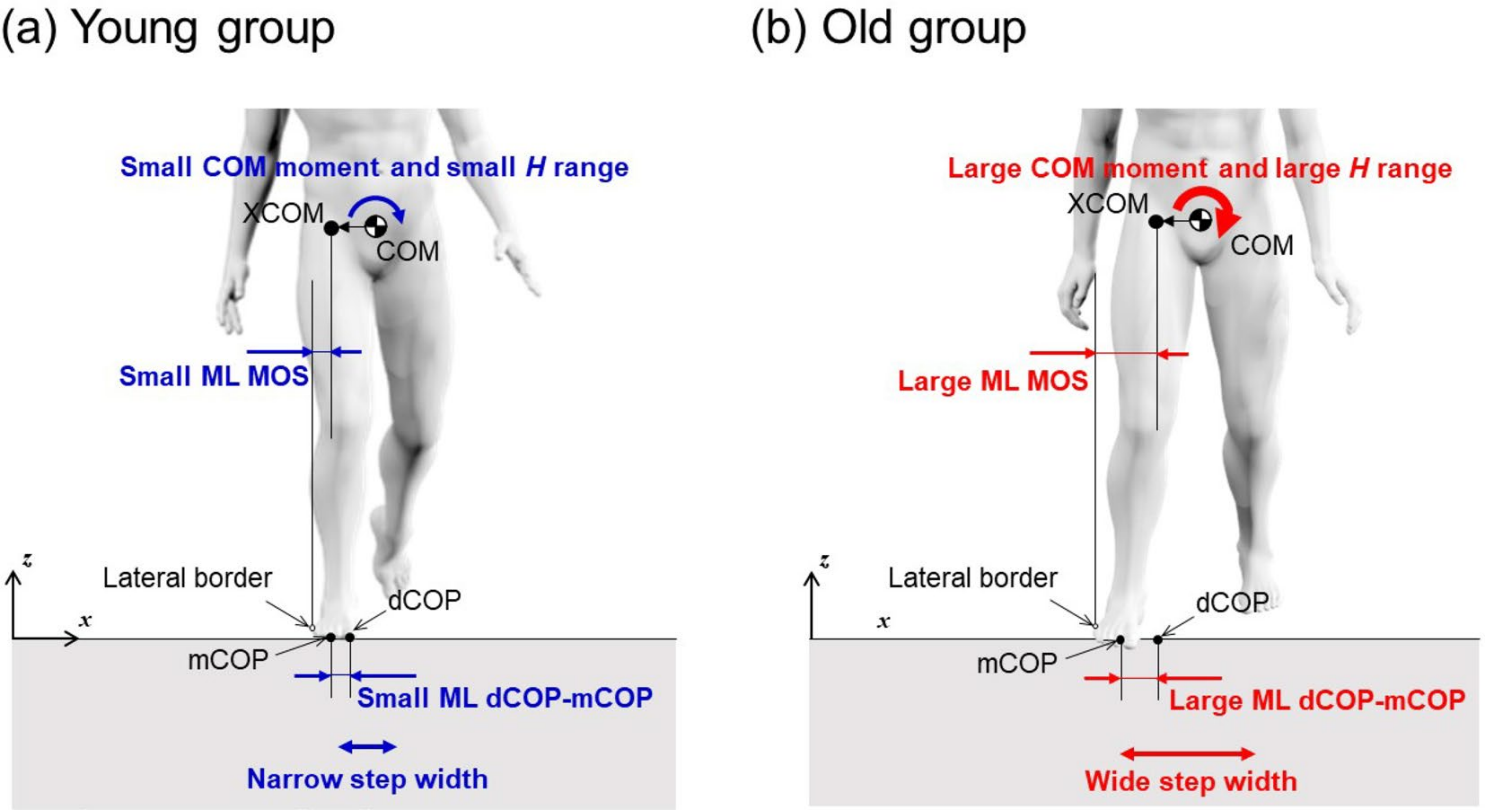
Schematic diagram of the difference in the relation between step width and dynamic balance measures in the ML direction between (**a**) young and (**b**) old groups. *ML* mediolateral.

Our results indicated that step width and all dynamic balance measures in the ML direction (i.e., MOS, peak-to-peak range of *H*, and peak dCOP–mCOP) were positively correlated with age (Figs. 1 and 2). The step width, stride time, and step time were affected by age group during walking: the old group walked with a wider step width than the young and middle-aged groups, and the middle-aged group walked with shorter stride and step time than the other age groups. All dynamic balance measures in the ML direction were greater in the old group than in the young group, whereas no significant differences were found between the age groups for balance measures in the AP direction. Thus, dynamic balance measures in the ML direction indicate age-related changes in balance during walking. These results are summarized as a schematic diagram in Fig. 3. The frontal peak-to-peak range of *H* was positively correlated with ML MOS and ML peak dCOP–mCOP.

### Effect of age on dynamic balance measures

Dynamic balance measures in the ML direction increased with increasing age across the lifespan (Fig. 2), and the old group walked with a greater MOS, range of *H*, and dCOP–mCOP in the ML direction (Table 1 and Fig. 3). ML MOS was positively correlated with step width (Table 2). MOS indicates the margin of XCOM to the BOS; hence, a larger MOS signifies a lower chance of XCOM moving out of the BOS. Thus, individuals in the old group probably took a strategy of increasing step width, by which they successfully increased the ML MOS. This result was also observed in a study comparing ML MOS between young and old adults during walking^17^, stepping on targets^18^, and head turning while walking^19^, indicating that ML MOS for older adults^18,19^ or older fallers^17^ are more compared with young adults due to a wider foot placement. The adaptive strategy of increasing step width probably increases the moment around the COM, as reflected in the increase of dCOP–mCOP, resulting in increased momentum around the COM captured by *H*. However, the range of *H* and peak dCOP–mCOP in the ML direction are affected more by age than by foot placement, unlike MOS in the ML direction (Table 2). This suggests that the range of *H* and ML peak dCOP–mCOP could be affected by other factors, such as a reduced lower limb joint moment. Reduced hip abduction moments due to muscle weakness^28^ may be subjected to increased ML dCOP–mCOP instead of or in addition to wide foot placement, resulting in the increased frontal range of *H* for older adults. Older adults generate lower ankle plantarflexor moment^29–31^, which plays a critical role in regulating whole-body angular momentum in both the sagittal^29^ and frontal^30^ planes during walking. Thus, the age-related reduction of the ankle plantarflexor moment may also be a factor that increased the ML dCOP–mCOP and frontal range of *H* in older adults. In general, a larger moment and momentum may destabilize the system in the sense that excessive moment/momentum results in falls. The range of *H* in the frontal plane is inversely correlated with clinical balance scores, such as the Berg balance scale and dynamic gait index in patients with stroke^15^. This suggests that people who have poor balance with increased moment/momentum about the COM may take a strategy of increasing step width^32^ as a proactive balance recovery action, resulting in increased MOS.

No correlations were found between age and any dynamic balance measures in the AP direction (Fig. 2), and no significant differences were found between age groups in any dynamic balance measures in the AP direction (Table 1). Moreover, gait parameters in the AP direction were correlated with dynamic balance measures, as shown in Table 2: AP MOS was negatively correlated with step length and positively correlated with step velocity, AP range of *H* positively correlated with step length, and AP peak dCOP–mCOP was negatively correlated with stride velocity. As these gait parameters were not affected by age (Table 1), it is reasonable that there were no age-related changes in the AP MOS, AP range of *H*, or AP peak dCOP–mCOP.

### Correlations between dynamic balance measures

In the ML direction, the correlations between the MOS and range of *H,* and between the range of *H* and peak dCOP–mCOP were moderate, whereas the correlation between the MOS and dCOP–mCOP was not significant (Table 3). Considering the dynamics, a moderate correlation between *H* and dCOP–mCOP is reasonable, as greater moments about the COM must induce greater momentum^13^. Interestingly, the range of *H* was correlated with MOS, whereas dCOP–mCOP was not, although there was a correlation between the range of *H* and peak dCOP–mCOP (Table 3). As mentioned above, the MOS and range of *H* in the ML direction can be related to one another based on gait dynamics, and the moderate correlation between them can be accounted for by this. By comparison, no correlation was found between ML dCOP–mCOP and step width (Table 2), whereas ML dCOP–mCOP showed a correlation with step velocity (*β* = 0.306, *p* < 0.01). This may be due to other factors than foot placement, such as age-related reduction in the lower limb joint moment, which includes hip abduction and plantarflexor moments in older adults. Therefore, the MOS that was correlated strongly with step width was not necessarily relevant for dCOP–mCOP.

In conclusion, using a large sample data, we demonstrated that dynamic balance measures, such as the MOS, range of *H*, and peak dCOP–mCOP in the ML direction, were able to indicate an age-related reduction in dynamic balance in the ML direction during walking. Our results indicated that older adults walked with a greater step width, resulting in a large MOS in the ML direction. However, momentum and moment around the COM in the frontal plane was more affected by age than by foot placement, unlike MOS. Further studies are warranted to investigate the difference in age effect on each dynamic balance measure.

## Methods

### National institute of advanced industrial science and technology gait database

We used the publicly available National Institute of Advanced Industrial Science and Technology (AIST) Gait Database 2015^23^. This includes kinetic and kinematic data of level, straight gait for 214 Japanese individuals aged 7–77 years. The individuals lived independently in local communities, and they were able to walk independently without assistive devices. The experimental protocol was approved by the local Institutional Review Board of the National Institute of Advanced Industrial Science and Technology (AIST), and all the participants provided written informed consent before participating in the study. All the methods and experiments were conducted following the relevant guidelines and regulations.

The experimental setup and protocol were as described elsewhere^23,33,34^. Briefly, gait trials were conducted on a straight 10-m walkway, in which six force plates (BP400600-2000PT; AMTI, Watertown, MA, USA) were installed to record the ground reaction force (GRF) components *F*_*x*_, *F*_*y*_, and *F*_*z,*_ for the ML, AP, and vertical directions, respectively. A 3D motion capture system (Vicon MX; Vicon Motion Systems, Oxford, UK) was used to measure full-body kinematics, with 55 infrared reflective markers attached under the guidelines for Visual 3D software (C-Motion, Rockville, MD, USA). The sampling frequencies for GRF and 3D motion data were 1 kHz and 200 Hz, respectively.

The participants were asked to walk barefoot at a comfortable, self-selected speed. They were allowed sufficient practice walks to ensure a natural gait. After practice, 10 successful trials were recorded. GRFs for three steps on the force plates were recorded. The kinetic and kinematic data were low-pass-filtered using a fourth-order Butterworth filter with zero lag and cut-off frequencies of 10 Hz and 6 Hz, respectively. Low-pass filtering and calculation of the center of pressure for each foot and the whole-body COM were performed using Visual 3D software.

### Data analysis

The kinetic and kinematic database data of 151 participants aged 20–77 years were used. We excluded the data of 26 participants who were younger than 20 years and of 37 participants whose GRF data for three steps was not successfully captured because of misstepping. The participants were divided into the following three age groups: young (n = 44; age, 20–34 years), middle-aged (n = 36; age, 35–64 years), and old (n = 71; age, 65–77 years). The mean ± standard deviation of age, height, and body mass of participants, respectively, were 25.4 ± 4.4 years, 1.63 ± 0.07 m, and 54.8 ± 11.7 kg in the young group; 51.6 ± 9.2 years, 1.65 ± 0.09 m, and 59.3 ± 10.9 kg in the middle-aged group; and 68.3 ± 3.0 years, 1.59 ± 0.08 m, and 59.0 ± 9.1 kg in the old group. Height differed significantly by age group (one-way ANOVA, *F*[2,148] = 7.925, *p* < 0.01); height in the old group was significantly shorter than that in the other age groups (*t*-test with Bonferroni correction, *p* < 0.05). No significant differences were found in body mass among the age groups (*t*-test with Bonferroni correction, *p* > 0.05).

For the analysis, we used the kinetic and kinematic data for 10 strides in 10 trials per participant. We used Matlab ver. 8.3 (Mathworks, Natick, MA, USA) to calculate stride length, step length, step width, stride velocity, step velocity, stride time, and step time, to investigate the effect of aging on spatiotemporal gait parameters, in addition to the dynamic balance measures.

#### Gait parameters

Stride length was calculated as the longitudinal distance (AP [*y*] direction) between the heel markers of the first and third stepping feet at the heel-strike event of each foot. Step length was the longitudinal distance between the heel markers of two successive stepping feet at heel contact. Step width was calculated as the lateral distance between the centers of the two feet (approximated as the midpoints between the toe and heel markers) at the heel-strike event of each foot^35^. Stride velocity was calculated as the stride length divided by the time between heel contacts of the first and third stepping feet (i.e., stride time). Step velocity was also calculated using step length and the time between heel contacts of two successive stepping feet (i.e., step time).

#### Dynamic balance measures

**Figure 4:**
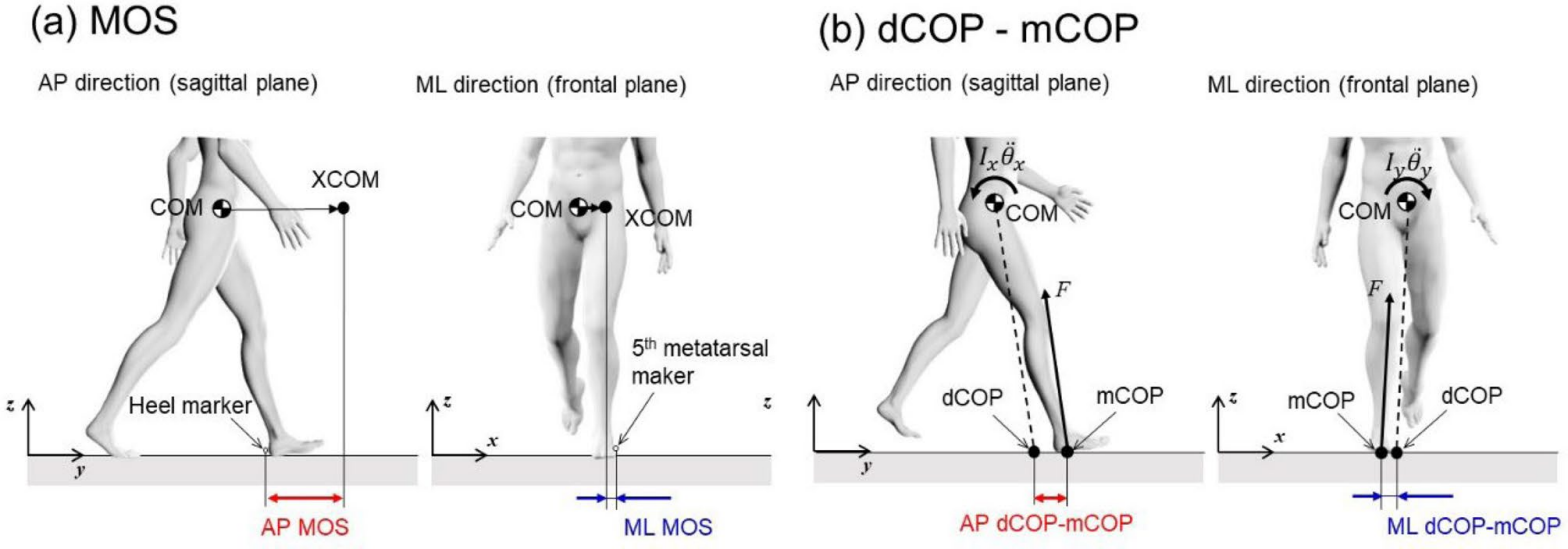
Schematic of the (**a**) margin of stability and (**b**) dCOP location with respect to mCOP in the AP and ML directions. *dCOP* desired center of pressure; *mCOP* measured center of pressure; *AP* anteroposterior; *ML* mediolateral.

**Figure 5:**
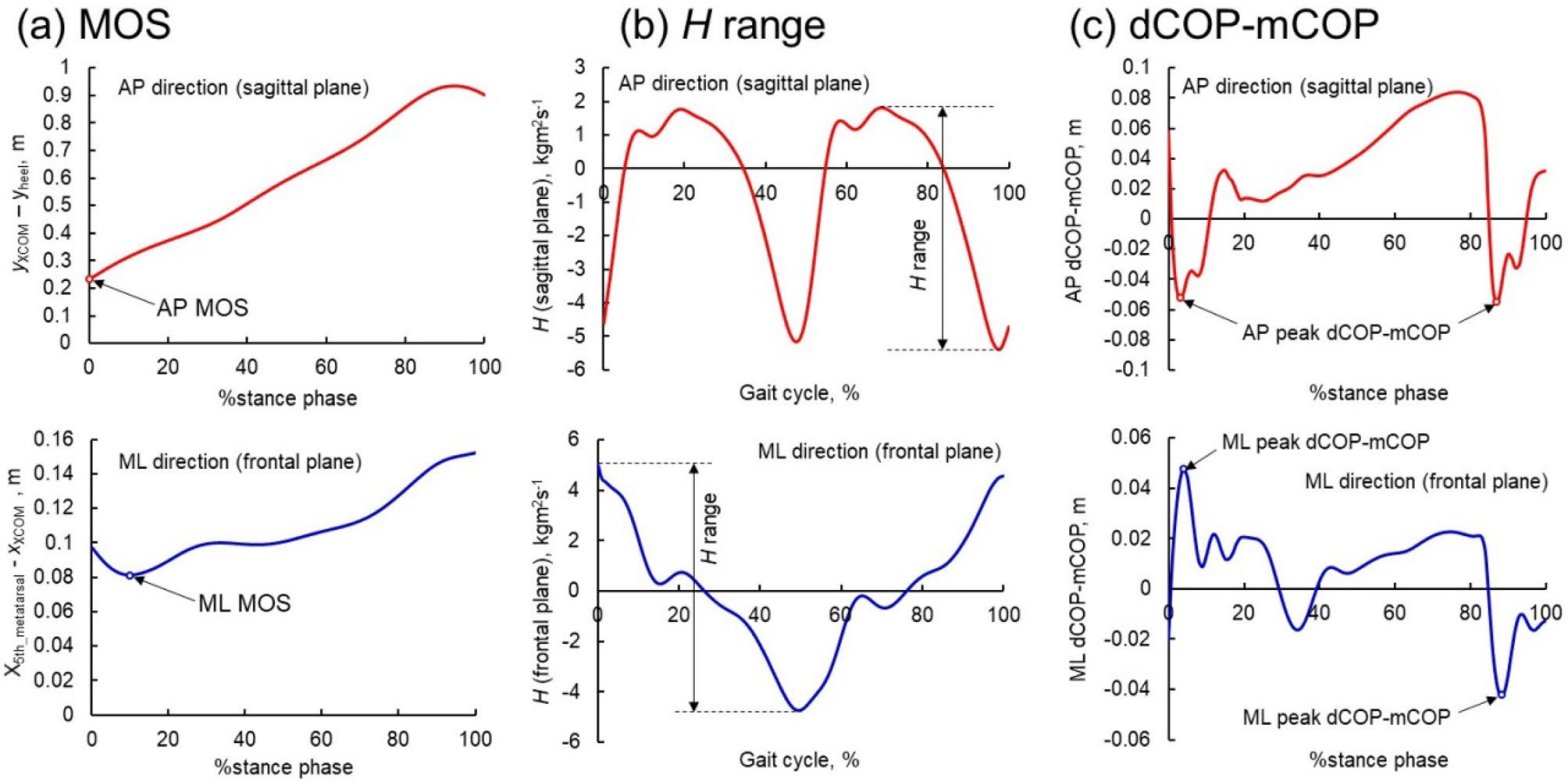
Representative time series for the (**a**) margin of stability, (**b**) range of *H*, and (**c**) dCOP–mCOP in the AP and ML directions. *H* whole-body angular momentum; *dCOP* desired center of pressure; *mCOP* measured center of pressure; *AP* anteroposterior; *ML* mediolateral.

AP MOS was defined as the distance in the AP direction between the XCOM and the posterior edge (heel marker) of the leading foot at heel contact (Figs. 4a and 5a)^36^. ML MOS was defined as the minimum distance in the ML direction between the XCOM and the lateral edge of the foot (fifth metatarsal marker) during the stance phase (Figs. 4a and 5a)^36^. XCOM in the ML (*x*_XCOM_) and AP directions (*y*_XCOM_) were calculated using the following equations^11^.

1 $$x_{XCOM} = x_{COM} + \frac{{\dot{x}_{COM} }}{{\sqrt {g/l} }}$$

2 $$y_{XCOM} = y_{COM} + \frac{{\dot{y}_{COM} }}{{\sqrt {g/l} }}$$

where *x*_COM_ and *y*_COM_ are the body COM position and $\dot{x}_{{{\text{COM}}}}$ and $\dot{y}_{{{\text{COM}}}}$ are the COM velocity in the ML and AP directions, respectively. *g* was the gravitational acceleration (9.81 m/s^2^), and *l* is the equivalent pendulum length, which is the length between the ankle and the COM.

*H* was calculated using the following equation^13,14^.

3 $${\mathop{H}\limits^{\rightharpoonup}} = \sum\limits_{i = 1}^{12} {\left[ {\left( {{\mathop{r}\limits^{\rightharpoonup}}{} _{i}^{COM} - {\mathop{r}\limits^{\rightharpoonup}}{} _{body}^{COM} } \right) \times \left( {{\mathop{v}\limits^{\rightharpoonup}}{} _{i}^{COM} - {\mathop{v}\limits^{\rightharpoonup}}{} _{body}^{COM} } \right) + I_{i} {\mathop{\omega }\limits^{\rightharpoonup}}{} _{i} } \right]}$$

where ${\mathop{r}\limits^{\rightharpoonup}} {}_{i} ^{COM}$ and ${\mathop{\nu}\limits^{\rightharpoonup}} {}_{i} ^{COM}$ are the position and velocity vectors, respectively, of the *i*th segment’s COM. ${\mathop{r}\limits^{\rightharpoonup}} {}_{body}^{COM}$ and ${\mathop{v}\limits^{\rightharpoonup}} {}_{body}^{COM}$ are the position and velocity vectors, respectively, of the whole-body COM. $\mathop{\omega }\limits^{\rightharpoonup} _{i}$, $m_{i}$, and $I_{i}$ are the angular velocity vector, mass, and moment of inertia of the *i*th segment, respectively. We used a 12-segmental model consisting of head, trunk, upper arms, forearms, thighs, shanks, and feet. The peak-to-peak range of *H* in the sagittal and frontal planes over the entire gait cycle were used for analysis (Fig. 5b).

The dCOP location in the ML (*x*_dCOP_) and AP directions (*y*_dCOP_) was expressed as^16^:

4 $$x_{dCOP} = x_{COM} - \frac{{F_{x} }}{{F_{z} }}z_{COM}$$

5 $$y_{dCOP} = y_{COM} - \frac{{F_{y} }}{{F_{z} }}z_{COM}$$

where *z*_COM_ is the whole-body COM location in the vertical direction. When the mCOP departs from dCOP, the misalignment (dCOP–mCOP) produces non-zero external COM moments ($I_{x} \ddot{\theta }_{x}$, and $I_{y} \ddot{\theta }_{y}$ in the sagittal and frontal planes, respectively) (Fig. 4b). The peak values of dCOP–mCOP during the weight acceptance phase for successive leading feet (appearing at approximately 0–5% stance phase and 90% stance phase, i.e., just after the heel contact of two successive steps) in the ML and AP directions were used for analysis (Fig. 5c). In the ML direction, the absolute magnitude of the peak value of dCOP–mCOP was used.

### Statistical analysis

Pearson correlation tests were performed to investigate correlations between age and gait parameters/balance measures. Age group differences in spatiotemporal gait parameters and balance measures were evaluated using ANCOVA, with body height as a covariate to mitigate the effects of age-related change in body height. When body height was not determined as a covariate, we performed one-way ANOVA. A post-hoc *t*-test with Bonferroni correction was used to determine specific significant differences between age groups. The effect size in terms of Cohen’s *d* for post-hoc *t*-tests was also reported. A Cohen’s *d* of 0.2–0.4, 0.4–0.8, and > 0.8 indicated small, moderate, and large effects, respectively^37^.

A stepwise multiple regression analysis was performed to describe the relationship between the independent variables (age, body height, body mass, and gait parameters) and dependent variables (dynamic balance measures). Partial correlation coefficients between two dynamic balance measures (adjusting for the other dynamic balance measures) were also computed to investigate relationships among the dynamic balance measures. All statistical analyses were performed using IBM SPSS Statistics for Windows, Version 19.0 (IBM Corp., Armonk, NY, USA). A *p-*value < 0.05 was considered to indicate significance.

## Data Availability

The data that support the findings of this study are available from the corresponding author upon reasonable request.
